# Genetic Association Study and Machine Learning to Investigate Differences in Platelet Reactivity in Patients with Acute Ischemic Stroke Treated with Aspirin

**DOI:** 10.3390/biomedicines10102564

**Published:** 2022-10-13

**Authors:** Anna Ikonnikova, Anastasia Anisimova, Sergey Galkin, Anastasia Gunchenko, Zhabikai Abdukhalikova, Marina Filippova, Sergey Surzhikov, Lidia Selyaeva, Valery Shershov, Alexander Zasedatelev, Maria Avdonina, Tatiana Nasedkina

**Affiliations:** 1 Engelhardt Institute of Molecular Biology, Russian Academy of Sciences, 119991 Moscow, Russia; 2 Department of Neurology, Neurosurgery and Medical Genetics, Faculty of Medicine, Pirogov Russian National Research Medical University, Ministry of Health of the Russian Federation, 117997 Moscow, Russia

**Keywords:** aspirin resistance, genetic markers, genetics, machine learning, CatBoost, ischemic stroke, SNP, pharmacogenetics, platelet aggregation, biochip

## Abstract

Aspirin resistance (AR) is a pressing problem in current ischemic stroke care. Although the role of genetic variations is widely considered, the data still remain controversial. Our aim was to investigate the contribution of genetic features to laboratory AR measured through platelet aggregation with arachidonic acid (AA) and adenosine diphosphate (ADP) in ischemic stroke patients. A total of 461 patients were enrolled. Platelet aggregation was measured via light transmission aggregometry. Eighteen single-nucleotide polymorphisms (SNPs) in *ITGB3*, *GPIBA*, *TBXA2R*, *ITGA2*, *PLA2G7*, *HMOX1*, *PTGS1*, *PTGS2*, *ADRA2A*, *ABCB1* and *PEAR1* genes and the intergenic *9p21.3* region were determined using low-density biochips. We found an association of rs1330344 in the *PTGS1* gene with AR and AA-induced platelet aggregation. Rs4311994 in *ADRA2A* gene also affected AA-induced aggregation, and rs4523 in the *TBXA2R* gene and rs12041331 in the *PEAR1* gene influenced ADP-induced aggregation. Furthermore, the effect of rs1062535 in the *ITGA2* gene on NIHSS dynamics during 10 days of treatment was found. The best machine learning (ML) model for AR based on clinical and genetic factors was characterized by AUC = 0.665 and F1-score = 0.628. In conclusion, the association study showed that *PTGS1*, *ADRA2A*, *TBXA2R* and *PEAR1* polymorphisms may affect laboratory AR. However, the ML model demonstrated the predominant influence of clinical features.

## 1. Introduction

Aspirin is a key drug widely used for ischemic stroke patients as antiplatelet therapy to prevent recurrent ischemic events [1]. This drug acts by irreversibly blocking the activity of the cyclooxygenases (COX)-1 and -2 also known as prostaglandin G/H synthases 1 and 2 (PTGS1 and PTGS2), respectively [2]. While the COX-1 enzyme is produced constitutively, the COX-2 form is highly inducible, mainly by inflammation. The COX-1 enzyme is expressed in mature platelets and catalyzes the conversion of arachidonic acid (AA) to prostaglandins G2 and H2, with a subsequent production of thromboxane A2 (TXA2) [3,4]. Thromboxane A2 is released into the bloodstream and binds to TXA2 receptors on the surface of neighboring platelets, causing their activation. Additionally, TXA2 acts synergistically with other substances released by activated platelets (adenosine diphosphate (ADP), fibrinogen, factor V) to increase the process. The main antithrombotic effect of low-dose (75–125 mg) aspirin is mediated by selective inhibition of COX-1 [5]. As a result of aspirin action, the production of TXA2, which is the main compound in platelet activation and aggregation, is suppressed for the lifetime of the platelet (7–10 days) [2]. The pathway of TXA2 production and the antiplatelet effect of aspirin are shown in Figure S1.

The response to aspirin varies between individuals, and up to 57% of patients show the so-called aspirin resistance (AR) [6]. AR is classified into clinical and laboratory resistance. Clinical AR is established by the inability of aspirin to prevent the subsequent acute vascular events [7]. Laboratory AR can be defined as ex vivo high on-treatment platelet reactivity (HTPR) such as the insufficient antiplatelet effect of aspirin measured by different laboratory tests [7,8]. Tests measure inactive metabolites of TXA2 in serum or urine [9,10] or analyze platelet aggregation and adhesion. Among the assays that determine platelet function, light transmission aggregometry (LTA) is considered as the gold standard in platelet function testing [11]. Automated (point-of-care) assays such as VerifyNow^®^, PFA-100^®^, Multiplate^®^, Plateletworks^®^ and others are widely used for monitoring platelet response to antiplatelet agents including aspirin [6,12,13]. HTPR was shown to increase the risk of recurrent vascular events and long-term clinical outcomes for patients with cerebrovascular pathology [14,15,16,17]. Nevertheless, platelet function tests differ in their ability to predict the risk of cardiovascular outcomes [12].

AR seems to be a complex phenomenon with a number of factors potentially contributing to it, but its causes and mechanisms are still unclear [18]. One of these factors that might underlie AR is heredity, having a profound impact on the variability in residual platelet function during aspirin therapy [19]. Genes encoding key platelet aggregation proteins are under the most intense scrutiny.

A number of genetic markers have already been studied to assess their possible contribution to AR [6,20]. First, single-nucleotide polymorphisms (SNPs) in the genes encoding COX enzymes (*PTGS1* and *PTGS2*) were found to influence AR [21,22,23,24,25,26,27,28]. Polymorphisms in the *TBXA2R* gene, encoding the specific TXA2 receptor, were associated with the effect of aspirin in a number of studies [29,30,31]. The genes involved in the COX-independent platelet activation pathways as well as platelet glycoprotein genes might also be involved in AR. The effect of polymorphisms in the genes *HMOX1* [24], *PLA2G7* [30], *ADRA2A* [30], *ITGB3* [22,32], *GPIBA* [33], *ITGA2* [34] and *PEAR1* [35,36,37] on inter-individual variations in the aspirin response has been discussed. A locus on chromosome *9p21.3*, associated with CVD and ischemic stroke, was also connected with AR [30,38]. P-glycoprotein (also known as MDR1) plays a crucial role in the intestinal epithelial cell permeability to aspirin [39] and might be involved in aspirin absorption. The TT rs1045642 genotype in the gene *ABCB1* encoding P-glycoprotein was shown to protect against AR [29]. Therefore, the molecular changes in the pathways involving various genes appear to influence the AR development. However, the impact of genetic markers on the risk for an individual patient is poorly understood. Implementing the identified genetic risk factors to predict aspirin failure in clinical practice still remains challenging.

One problem lies in the inconsistency of the results from genetic studies. This may be explained by the differences in the diagnoses (ischemic stroke, cardiovascular disease, diabetes mellitus), ethnic groups, platelet function tests, sample sizes, etc. [6]. There is a noticeable lack of replication studies analyzing AR genetic background in patients with ischemic stroke from the Eastern European populations.

Another problem is the multiplicity of influencing factors that determine the ultimate success or failure of aspirin therapy. The clinical features of the disease, comorbidities, co-medications and non-modifiable risk factors such as age should be taken into account [6]. Moreover, the interaction of genetic polymorphisms as well as clinical factors may influence sensitivity to aspirin [40]. Over the past several years, machine learning (ML) models have been proven to be able to solve various problems in the medical and biological fields, including pharmacogenetics [41,42]. One of the key advantages of the ML approaches lies in their ability to find unobvious relationships and make inferences from the complex data.

The purpose of this study was to investigate genetic features associated with laboratory AR in a cohort of patients with ischemic stroke taking aspirin as antiplatelet therapy to be used in pharmacogenetic testing. We have developed a biochip assay to identify 18 SNPs previously described as markers affecting AR. To establish the connection between the patients’ clinical data, genotype and laboratory response to aspirin treatment, we applied the multiple ML approaches.

## 2. Materials and Methods

### 2.1. Patients

The study included 461 Caucasian patients with primary ischemic stroke treated in the Stroke Center of City Clinical Hospital No.1 named N.I. Pirogov. The inclusion criterion was a verified ischemic stroke. Exclusion criteria comprised hemorrhagic transformation, cancer and severe liver disease, as well as other diseases and conditions affecting the parameters of platelet hemostasis. The pathogenetic variant of stroke was established according to the TOAST criteria [43] based on the clinical data, computed tomography and magnetic resonance imaging of the brain, Doppler ultrasound of the cerebral arteries and electrocardiography. The study population included 109 patients with cardioembolism, 98 patients with large artery atherosclerosis (LAA, ≥50% stenosis) and 250 patients with undetermined etiology (of which 53 had both LAA and cardioembolism, 197 had neither LAA nor cardioembolism). All patients received the antiplatelet, lipid-lowering, antihypertensive or anticoagulant therapy according to the clinical guidelines. For early prevention of recurrent stroke, all patients took aspirin at a dose of 125 mg daily, starting within 24 h of the stroke onset. Patients with cardioembolic stroke received the anticoagulant treatment starting on day 3, 6 or 12 depending on the stroke severity [44]. Dynamics of the NIHSS score estimated at admission and after 10 days of aspirin therapy was considered as the short-term clinical outcome.

The study was approved by the local ethics committee of the Pirogov Russian National Research Medical University (protocol no. 181 dated 28 January 2019). All participants provided a written informed consent. The study adhered to the World Medical Association Declaration of Helsinki. With a 95% confidence level, a standard deviation of 0.5 and a confidence interval (margin of error) of ±5%, the sample size was estimated to be 391 patients.

### 2.2. Platelet Aggregation

Blood samples from the vein of the non-paretic limb were collected in the morning of the third day of aspirin intake. The region of the cubital fossa was usually selected as the venipuncture area. A tourniquet was applied to the middle third of the shoulder, while the pulse was taken on the nearest radial artery. After that, the patient clenched the hand into a fist and unclenched it several times. The skin in the venipuncture area was stretched, fixing the vein. Next, the skin was pierced next to the vein; the needle was moved 1.5 cm deep into the subcutaneous fat, and the vein was punctured. A total of 9 mL of blood was collected in the 14 mL plastic test tubes “Greiner” with 1 mL of 3.8% trisubstituted sodium citrate using 21 G × 1 1/2”/0.8 × 40 mm needles. The blood in the tube was mixed immediately. Stabilized blood was stored at room temperature for no more than 30 min prior to centrifugation. The samples were centrifuged at 200× *g* for 7 min. Then, 2.5 mL of the supernatant containing platelet-rich plasma was carefully taken for analysis in the aggregometer. Platelet aggregation was measured by LTA using the laser analyzer of platelet aggregation ALAT-2 (Biola Scientific, Moscow, Russia) based on the method of Born and O’Brien.

To identify a group of patients with AR, we relied on the criteria proposed by Gum et al. [45]. AR was defined as aggregation of ≥70% with 10 μm ADP and aggregation of ≥20% with 0.5 mM AA. Aspirin semi-resistance (ASR) was defined as aggregation of ≥70% with 10 μM ADP or aggregation of ≥20% with 0.5 mM AA [45]. The patients with AR and ASR were pooled into the AR group. Patients with ADP-induced aggregation <70% and AA-induced aggregation <20% were considered aspirin-sensitive (AS) and were assigned to the AS group [22].

### 2.3. DNA Extraction

Genomic DNA was extracted from the blood collected into the EDTA-containing tubes using the QIamp DNA Mini kit (Qiagen, Hilden, Germany) according to the manufacturer’s instructions. DNA was isolated from 200 μL of the whole blood. The procedure included cell lysis, sorption on the silica gel membrane of the column, washing and elution (in 100 µL of elution buffer). The DNA concentration was measured using the NanoDrop 1000 spectrophotometer (Thermo Fisher Scientific, Waltham, MA, USA). DNA samples were subjected to further analysis if DNA concentration was at least 10 ng/µL and its 260/280 ratio was in the range of 1.75 to 1.95.

### 2.4. Selection of SNPs and Genotyping

Genetic markers in ten genes (*ITGB3*, *GPIBA*, *TBXA2R*, *ITGA2*, *PLA2G7*, *HMOX1*, *PTGS1*, *PTGS2*, *ADRA2A*, *ABCB1*, *PEAR1*) and one intergenic region (*9p21.3*) were selected (Table 1).

Genotyping involved the multiplex one-step PCR followed by allele-specific hybridization on a biochip as described before [46]. 2′-deoxyuridine 5′-triphosphate (dUTP) derivatives containing the Cy7 cyanine dye were used as fluorophores [47]. The sequences of primers and allele-specific oligonucleotide probes are listed in the Supplementary Tables S1 and S2. The biochip scheme and an example of the hybridization picture are shown in Figure S2. Genotyping results were verified by direct sequencing and high-resolution melting analysis.

### 2.5. Statistical Analysis

The online service SNPStats (https://www.snpstats.net/ (accessed on 26 April 2022)) [48] was used to evaluate the association of genotypes with aspirin resistance and aggregation with AA and ADP as well as the NIHSS score dynamics (adjusted by clinical variables). We used individual SNPs’ data for co-dominant, dominant, recessive and log-additive models. Comparison of baseline characteristics in groups with different genotypes was performed using the Kruskal–Wallis test and the chi-square test. Allele frequencies between AS and AR groups were compared using the two-sided Fisher exact test. Statistical analysis was performed in R (version 4.1.1; R Foundation for Statistical Computing, Vienna, Austria). The differences were considered statistically significant if the *p*-value was below 0.05. The boxplots display the median, two hinges which correspond to the first and third quartiles and two whiskers. The upper and lower whiskers extend from the hinges to the largest value no further than 1.5×IQR from the corresponding hinge (where IQR is the inter-quartile range). Points beyond the whiskers indicate the outliers.

### 2.6. Machine Learning

**Figure 1:**
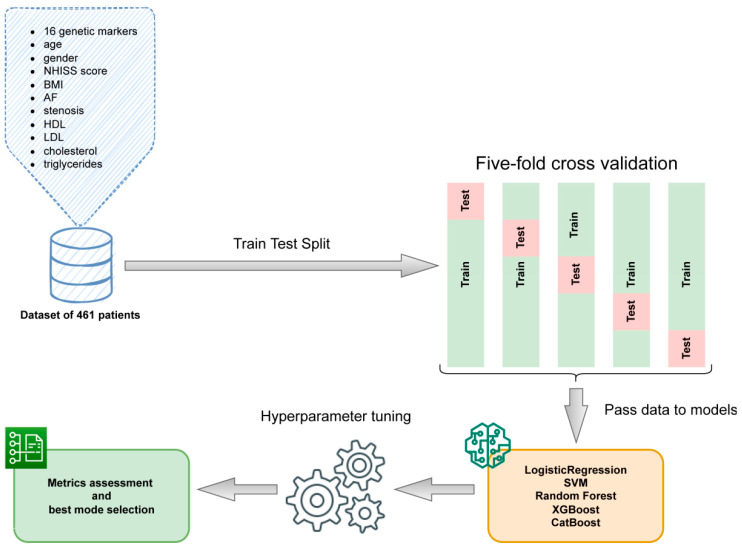
The machine learning pipeline.

To build a predictive machine learning model, several approaches have been tested using the following Python 3.8 libraries: sklearn.linear_model.LogisticRegression, sklearn.svm.SVC, sklearn.ensemble.RandomForestClassifier [49], XGBoost [50] and CatBoost [51]. All models were trained in a five-fold cross validation (CV) setting with folds stratified to keep the proportion of studies similar to the whole data set. Each model parameter was optimized in order to increase the classification metrics: accuracy, AUC and F1-score, paying the most attention to the latter metric. The array of features consisted of all 16 genetic markers along with the age, gender, NHISS score at admission, body mass index (BMI), atrial fibrillation (AF), stenosis, high-density lipoproteins (HDLs), low-density lipoproteins (LDLs), cholesterol and triglycerides. Feature importance ranking was obtained using Shapley additive explanations (SHAP) values, a game theoretic approach to explain the output of any machine learning model [52]. The sequence of the ML procedure pipeline is shown in Figure 1.

## 3. Results

### 3.1. Baseline Characteristics of AR and AS Patients

The baseline clinical characteristics of patients are shown in Table 2. A total of 461 patients were included in the analysis. Full AR and ASR were established in 28 patients (6.1%) and 192 patients (41.6%), respectively, and these two groups were pooled into one AR group. Another 241 patients (52.3%) were AS.

The AS and AR groups differed in some clinical parameters (Table 2). AR patients were significantly older: the mean age of 73.21 years in the AR group vs. 68.72 years in the AS group (*p* < 0.001). AR patients had a more severe stroke: the mean NHISS score at admission was 12.35 and 10.45 in the AR and AS group, respectively (*p* < 0.001). Moreover, atrial fibrillation was more frequent in the AR group (41.74%) compared to the AS group (29.58%) (*p* = 0.0088).

### 3.2. The Association of SNPs with Aspirin Resistance in Whole Cohort of Patients

A total of 461 samples were genotyped for selected SNPs (Table 3). Genotype frequencies in the total sample, AR and AS groups conformed to the Hardy–Weinberg equilibrium (data not shown). The rs1126643 and rs1062535 markers in the *ITGA2* gene as well as rs1051931 and rs7756935 in the *PLA2G7* gene were in strong linkage disequilibrium (D’ = 1.0, R2 = 1.0), and only one of them in each pair was included in the analysis.

Allele and genotype frequencies for sixteen SNPs in the AS and AR groups are listed in Table 3. The frequency of the minor allele C for rs1330344 *PTGS1* was significantly higher in the AR group than in the AS group (27% vs. 21%, *p* = 0.044).

The association of genotypes with the response to aspirin was investigated using the SNPStats online service. We included age, AF and the NHISS score at admission as covariates in the analysis, since they showed a different distribution between AR and AS groups (Table 2). The results for all studied markers are in Supplementary Table S1 (for AR and AS groups) and Supplementary Table S2 (for AA- and ADP-induced aggregation). We revealed the following associations.

The CC genotype of rs1330344 in the *PTGS1* gene was more frequent in the AR group than in the AS group (OR = 2.75, 95% CI = 1.14–6.63, *p* = 0.019). Data are shown in Supplementary Table S3.

**Figure 2:**
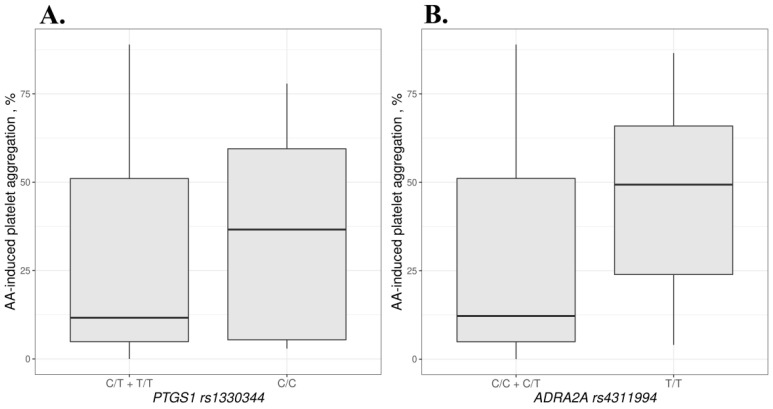
AA-induced aggregation based on the *PTGS1* rs1330344 (**A**) and *ADRA2A* rs4311994 (**B**) genotypes.

We compared the association of different genotypes with AA- and ADP-induced aggregation. For *PTGS1* rs1330344, mean AA-induced aggregation was 40.5% higher in the CC genotype compared to the TT + CT genotypes (*p* = 0.038). For rs4311994 in the *ADRA2A* gene, mean AA-induced aggregation was 72.7% higher in patients with the TT genotype compared to the CC + CT genotypes (*p* = 0.043). Data are shown in Figure 2 and in Supplementary Table S4.

**Figure 3:**
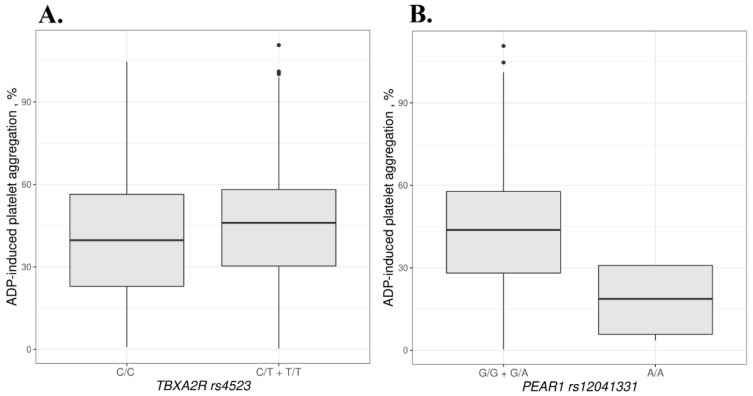
ADP-induced aggregation based on the *TBXA2R* rs4523 (**A**) and *PEAR1* rs12041331 (**B**) genotypes. Dots beyond the whiskers indicate the outliers.

Mean ADP-induced aggregation was 9.2% higher in the TT + CT genotypes of *TBXA2R* rs4523 compared to the CC genotype (*p* = 0.043). For rs12041331 in the *PEAR1* gene, mean ADP-induced aggregation was 59.5% lower in AA homozygotes compared to the GG + GA genotypes (*p* = 0.017). Data are shown in Figure 3 and in Supplementary Table S2.

### 3.3. The Association of SNPs with AR and Platelet Reactivity in Patients with Noncardioembolic Ischemic Stroke

In total, 296 patients had noncardioembolic ischemic stroke, with 127 (43%) and 169 (57%) patients being assigned to the AR and AS groups, respectively. We analyzed the frequency of different genotypes for sixteen SNPs in the AR and AS groups. Although the CC genotype of rs1330344 in the *PTGS1* gene was more frequent in the AR group than in the AS group (OR = 2.48, 95% CI = 0.93–6.60, *p* = 0.062), the difference is not statistically significant.

The CC homozygotes of *PTGS1* rs1330344 had 55.4% higher mean AA-induced aggregation compared to the TT + CT genotypes (*p* = 0.026). Mean ADP-induced aggregation was 14.8% higher in the TT + CT genotypes of rs4523 *TBXA2R* than in the CC genotypes (*p* = 0.031) and 11.6% lower in the AA + GA genotypes of *ITGA2* rs1062535 comparing to GG homozygotes (*p* = 0.051).

Note that *p*-values are given before the correction for multiple comparisons; after the Bonferroni correction, all *p*-values were >0.05.

### 3.4. Clinical Outcome Evaluation

We evaluated the clinical outcomes of patients with noncardioembolic ischemic stroke within the first 10 days after admission and their association with aspirin resistance. Five patients died and were excluded from the analysis. The NIHSS score on day 10 was compared with the admission NIHSS score. We found no statistically significant association of the NIHSS score dynamics analyzed with the groups of AR and AS patients.

Furthermore, the NIHSS score dynamics was evaluated in patients with different genotypes adjusted by age and the NIHSS score at admission. The AA + GA genotypes of rs1062535 in the *ITGA2* gene had worse dynamics in the NIHSS score compared to the GG genotype (5.3 vs. 6.57, *p* = 0.0008).

### 3.5. Machine Learning Model

To investigate the contribution of clinical and genetic features to AR, we created ML models. The overall best performance was achieved after utilizing CatBoost algorithm, high-performance open-source library for gradient boosting on decision trees. The parameters of the model that showed best performance in CV are listed in the Appendix A. For ML model generation, the total cohort of patients with ischemic stroke was included.

The ML models did not have enough predictive power if they were based only on genetic features. To overcome this limitation, we included anthropometric and clinical data in the model.

After training several models in a five-fold cross-validation setting, we compared the output metrics in order to choose the classification method with the best performance. As expected, the gradient boosting on the decision tree algorithm, CatBoost, outperformed logistic regression, the support vector machine and random forest classifiers since it was designed to leverage the information gained from categorical features. The average values of classification metrics were as follows: AUC = 0.665, F1-score = 0.628, specificity = 0.773, sensitivity = 0.60, precision = 0.63. The ML model is in the Supplementary Files (Model S1).

**Figure 4:**
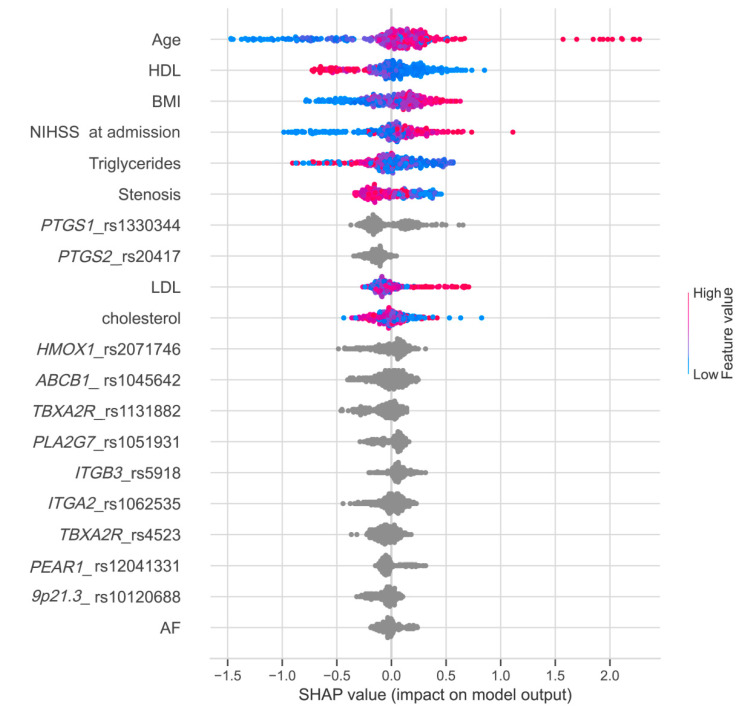
Feature importance ranking obtained using SHAP values. Variables are listed in order of significance from top to bottom on the *y*-axis. Each point represents a patient, and its color indicates the value of corresponding variable. The position of the points on the *x*-axis represents SHAP values, indicating the changes in log odds, and the probability of success can be extracted from this value.

To assess the impact of each feature on the model performance and identify the most important factors, we conducted the Shapley additive explanations analysis (Figure 4), which allowed us to study the relationships between variables for the predicted case and their contribution to the final score. Shapley values indicate the importance of a feature by comparing model predictions with and without this feature.

## 4. Discussion

In the present study, we used a biochip-based assay to analyze 18 SNPs in patients with acute ischemic stroke and variable response to aspirin treatment. The SNPs were selected based on the literature data. All of them are involved in platelet activation and aggregation, and their contribution to aspirin resistance is discussed in numerous studies [6,21,22,23,24,25,26,27,28,29,30,31,32,33,34,35,36,37]. We evaluated the distribution of 16 genetic markers in the AS and AR groups in a cohort of 461 patients with acute ischemic stroke.

The aspirin resistance was associated with the following clinical parameters: age, the NIHSS score at admission and atrial fibrillation (Table 2). Aging is known to be associated with an elevated platelet activity [53] as well as aspirin resistance [45,54], which is consistent with our results. The initial NIHSS score was also higher in the AR patients [55]. In several studies concerning AR, ischemic stroke patients with atrial fibrillation (cardioembolism) were excluded from the analysis since they had anticoagulant therapy prescribed earlier [21,22]. In our study, all patients with all stroke variants received aspirin at least for the first 3 days, while laboratory AR was estimated during this period. This allowed us to enroll all the patients in the study, which aimed at identifying the associations between genetic markers and aspirin non-sensitivity. In addition, we performed the association studies in a cohort of non-embolic patients and evaluated clinical recovery for 10 days based on the NIHSS score dynamics. Determining the prognostic genetic markers of AR in this group can be very helpful given that the long-term aspirin treatment is recommended for these patients.

Among 16 SNPs studied, four genetic variants showed a significant association with aspirin non-sensitivity in the whole cohort: *PTGS1* (rs1330344), *ADRA2A* (rs4311994), *TBXA2R* (rs4523) and *PEAR1* (rs12041331).

The C allele and CC genotype (rs1330344) of the *PTGS1* gene encoding COX-1 were associated with AR and a higher level of AA-induced aggregation. A similar observation was made in the study by Li et.al. [23]. The CC genotype was associated with poor functional outcomes in Chinese patients with a stroke during aspirin therapy [40,56]. However, the obtained results were not always consistent [24,30,57,58]. This polymorphism is located in the regulatory region (T-1676C), and this substitution may lead to an increase in COX-1 activity and contribute to a decreased or absent response to aspirin [6]. The C allele was also found to be associated with an increased risk of ischemic stroke in the Chinese population [59]. However, in our study, rs10306114 in this gene, most frequently associated with AR [6], showed no association with AR.

Another polymorphism that demonstrated an association with AA-induced aggregation in our study, rs4311994, is also located in the regulatory region downstream (63 kb) of the 3’ end of the *ADRA2A* gene; its effect may arise from the regulation of gene expression or linkage disequilibrium with the other variants. In our study, the minor allele T of the *ADRA2A* gene (rs4311994) was associated with a higher level of AA-induced aggregation. The *ADRA2A* gene encodes the alpha-2A-adrenergic receptor involved in epinephrine-induced platelet aggregation and shear-dependent platelet function. This allele was associated with increased platelet reactivity to aspirin in the population with type 2 diabetes mellitus. [30]. However, these results were not always reproducible [60].

Notably, the alleles of the *PTGS1* and *ADRA2A* genes, associated with AR and/or high AA-induced aggregation in our study, correlated with a reduced risk of complications from the gastrointestinal tract when taking aspirin in other studies [61,62,63]. This may indicate a role in stimulating platelet activity in carriers of these alleles.

The *TBXA2R* rs4523 (T924C) affected ADP-induced aggregation: the aggregation was higher in the TT and CT genotypes than in the CC genotype. In other studies, the TT homozygotes also showed increased platelet reactivity [64,65]. It is a synonymous nucleotide change that can affect splicing or mRNA stabilization and translation efficiency. Otherwise, this SNP may be in linkage disequilibrium with other clinically relevant polymorphisms [64,65]. The other SNP (rs1131882) in the *TBXA2R* gene showed no association with AR in our study.

The ADP-induced aggregation was affected by intronic rs12041331 in the *PEAR1* gene being lower in the AA homozygote as compared to the GG and GA genotypes. These data are consistent with some other studies [19,37,66,67]. The *PEAR1* gene encodes the type 1 membrane protein expressed in platelets and endothelial cells. Its phosphorylation appears to promote platelet aggregation [68,69]. The rs12041331 polymorphism results in a G to A substitution in intron 1 and was previously shown to be implicated in reducing *PEAR1* expression [19]. According to Faraday et al. [19], the major G allele of rs12041331 was associated with a higher platelet aggregation both in the presence and absence of aspirin treatment. Thus, the influence of the *PEAR1* gene may not be specific to the aspirin action. The AA genotype of *PEAR1* rs12041331 was shown to be associated with an increased response to ticagrelor in healthy people [70]. However, some studies revealed no such association for this SNP [71].

In patients with noncardioembolic stroke, the polymorphism *PTGS1* rs1330344 showed a significant association with AA-induced aggregation. Thus, *PTGS1* rs1330344 might be considered as the strongest predictor of laboratory AR among the analyzed SNPs, both in the whole cohort of ischemic stroke and noncardioembolic patients. The second genetic marker associated with laboratory AR in both cohorts was rs4523 in *TBXA2R* gene. The T allele acted as a risk factor for increased ADP-induced aggregation during aspirin treatment.

An ambiguous association for *ITGA2* rs1062535 was revealed in noncardioembolic patients. The *ITGA2* gene encodes the alpha chain of the platelet collagen receptor integrin α2β1 (glycoprotein IA/IIa, GPIa/IIa), which promotes an initial interaction between platelets and collagen with further platelet activation and aggregation. The A allele of rs1062535 was suggested to stimulate the protein expression and increase affinity to collagen, which in turn facilitated platelet reactivity. The A allele of *ITGA2* rs1062535 was significantly associated with reduced post-operative bleeding after cardiac surgery [72]. In our study, on the contrary, the AA + GA genotypes correlated with lower ADP-induced aggregation. In contrast, in previously published data, the A allele was considered as a possible risk factor for thromboischaemic events [73]. This suggestion is in agreement with our findings implying a strong relationship between the A allele and negative NIHSS dynamics in noncardioembolic patients.

Thus, the role of genetic factors underlying the inter-individual differences in aspirin action is of immense interest, but further research is required to understand how genetic data can be efficiently applied to personalized therapy. Different approaches, such as general multifactor dimensionality reduction (GMDR), were employed to study the potential contribution of multiple genetic factors along with the single-locus analysis [22].

We applied the ML method to predict the risk of AR development using clinical and genetic factors. This is the first attempt to bring in the ML approach to the analysis of genetics of AR. We obtained an AUC = 0.665 for our best model (Model S1, Figure 4). On the one hand, this value seems to be modest, but on the other hand, it is in agreement with the parameters of other models based on ML for multifactorial processes. For example, similar sensitivity and specificity values were obtained for antidepressants [74,75]. However, in those studies, these parameters were obtained only from the genetic factors, whereas in our study they mainly depended on clinical factors. The developed ML model may be considered as a first approximation aimed at dealing with the problem of AR prediction. The relevance of the developed model for clinical practice is still to be confirmed. We assume that further studies involving larger and more clinically uniform cohorts of patients are required to shed light on the genetic background contributing to the resistance to aspirin treatment. Another approach relies on searching for more relevant genetic markers utilizing throughput methods of genetic analysis such as the next-generation sequencing. The assessment of polygenic risk score might prove promising as well.

As there is an alternative to aspirin for secondary stroke prevention, such as dual antiplatelet therapy or ticagrelor [76], identifying patients with a predisposition to AR can be used for personalized therapy to reduce the risk of adverse events. However, it is possible that the risk alleles for AR might also be associated with platelet aggregation when taking other antiplatelet drugs requiring special attention for such patients.

The current study has several limitations. First, when choosing genetic polymorphisms, we relied on the published studies focusing on certain candidate genetic markers. Searching for more relevant genetic markers using such high-throughput methods of genetic analysis as next-generation sequencing may prove promising. Moreover, given the complex nature of aspirin resistance, the polygenic risk score may be introduced for identifying patients with a high risk of aspirin treatment failure. The second limitation is related to the size of the studied population. It seems to be large enough compared with other studies in the field [21,24,25,26,27,28,29,30,31,32,33,34]. However, clarifying the genetic background of aspirin treatment failure, which is affected by numerous clinical parameters and studied SNPs, requires further studies including larger and more clinically uniform cohorts of patients. The third limitation may be related to clinical outcome assessment. A number of studies confirmed an increased risk of adverse outcomes in patients with laboratory AR [14,15,16,17]. However, the underlying mechanism of a poor response to aspirin is still unclear. The ex vivo platelet reactivity tests do not always clearly correlate with the therapeutic effect of the drug [77]. Our study focused on analyzing laboratory AR, but the most important results can be obtained from the long-term follow-up of patients and assessing the influence of genetic and clinical factors and laboratory measurements of AR on clinical outcomes. Finally, in our study, we were not always able to take into account the potential impact of other drugs used by our patients, such as anticoagulants or statins, which are usually prescribed for the secondary prevention of a stroke. The drug–drug interactions as well as malabsorption or renal dysfunction could also affect aspirin pharmacokinetics or pharmacodynamics and thus lead to a number of subsequent pharmacological effects [78,79].

## 5. Conclusions

Early detection of aspirin resistance in ischemic stroke patients is important for timely prescription of other antiaggregant drugs when possible. Therefore, searching for predictive markers of aspirin treatment failure is of great importance. In our study, we revealed the association between clinical parameters (age, NIHSS score, atrial fibrillation), as well as SNPs in the *PTGS1*, *ADRA2A*, *TBXA2R* and *PEAR1* genes, and laboratory indicators of platelet activity in ischemic stroke patients taking aspirin for secondary stroke prevention. The ML model of AR in the studied cohort of patients showed the prevailing contribution of clinical parameters. However, we assume that the genetic factors are a promising predictor of aspirin resistance. The ML approach revealed the prospective future directions of predicting the risk of AR development. Further replication studies including more homogeneous groups of patients, the implementation of high-throughput genotyping technologies and development of risk-predictive models based both on clinical and genetic features may be considered as key steps towards better understanding aspirin resistance in patients with an ischemic stroke.

## Figures and Tables

**Table 1:** A list of studied genetic markers.

Gene	rs ID	Wild-Type Allele	Minor Allele	Protein
*ITGB3*	rs5918	T	C	Platelet glycoprotein IIIa/Integrin subunit-Beta3
*GPIBA*	rs2243093	T	C	Glycoprotein Ib platelet subunit alpha
	rs6065	C	T	
*TBXA2R*	rs1131882	C	T	Thromboxane A2 receptor
	rs4523	C	T	
*ITGA2*	rs1126643	C	T	GPIa/IIa- Integrin alpha 2
	rs1062535	G	A	
*PLA2G7*	rs1051931	C	T	Lipoprotein-associated phospholipase A2/ Plasma platelet-activating factor acetylhydrolase
	rs7756935	A	C	
*HMOX1*	rs2071746	A	T	Heme oxygenase 1
*PTGS1*	rs10306114	A	G	Prostaglandin G/H synthase 1 (cyclooxygenase-1)
	rs1330344	T	C	
*PTGS2*	rs20417	G	C	Prostaglandin G/H synthase 2 (cyclooxygenase-2)
	rs689466	T	C	
*ADRA2A*	rs4311994	C	T	Alpha-2A-adrenergic receptor
*9p21.3*	rs10120688	G	A	Intergenic
*ABCB1*	rs1045642	T	C	MDR1, ATP-binding cassette subfamily B member 1
*PEAR1*	rs12041331	G	A	Platelet endothelial aggregation receptor-1

**Table 2:** The clinical characteristics and laboratory parameters in the AS and AR groups.

Characteristics	AS Group (*n* = 241)	AR Group (*n* = 220)	*p*-Value
Age, (mean ± sd)	68.72 ± 14.56	73.21 ± 14.52	<0.001
Sex, *n* (%)			
women	117 (48.5%)	119 (54.1%)	0.235
Type of stroke according to TOAST criteria, *n* (%):			
LAA	55 (12.04%)	43 (9.41%)	0.0859
Cardioembolism	49 (10.72%)	60 (13.13%)	
Undetermined etiology (with LAA and Cardioembolism)	22 (4.81%)	31 (6.78%)	
Undetermined etiology (without LAA and Cardioembolism)	113 (24.73%)	84 (18.38%)	
NHISS score at admission, (mean ± sd)	10.45 ± 6.49	12.35 ± 6.67	<0.001
AF, *n* (%)	71 (29.58%)	91 (41.74%)	0.0088
Stenosis, % (mean ± sd)	11.53 ± 7.18	12.01 ± 7.15	0.5408
BMI, mmol/L (mean ± sd)	27.64 ± 4.83	28.17 ± 4.99	0.2219
HDL, mmol/L (mean ± sd)	1.18 ± 0.34	1.15 ± 0.35	0.3584
LDL, mmol/L (mean ± sd)	2.96 ± 0.97	2.98 ± 0.99	0.9437
Cholesterol, mmol/L (mean ± sd)	4.84 ± 1.24	4.84 ± 1.32	0.9882
Triglycerides, mmol/L (mean ± sd)	1.52 ± 1.15	1.47 ± 0.85	0.4851
Atherogenic coefficient (mean ± sd)	3.22 ± 1.1	3.37 ± 1.24	0.2309

AF—atrial fibrillation, BMI—body mass index, HDL—high-density lipoproteins, LDL—low-density lipoproteins, LAA—large artery atherosclerosis, sd—standard deviation.

**Table 3:** Genotype and allele frequencies in the AS and AR groups.

		AS Group					AR Group					
Gene	rs ID	wt, *n*	het, *n*	mut, *n*	Wild-Type Allele, %	Minor Allele, %	wt, *n*	het, *n*	mut, *n*	Wild-Type Allele, %	Minor Allele, %	*p*-Value *
*ITGB3*	rs5918	164	68	9	82	18	148	63	9	82	18	0.864
*GPIba*	rs2243093	167	68	6	83	17	154	61	5	84	16	0.859
*GPIba*	rs6065	211	29	1	94	6	182	37	1	91	9	0.173
*TBXA2R*	rs1131882	162	72	7	82	18	164	50	6	86	14	0.127
*TBXA2R*	rs4523	92	114	35	62	38	91	99	30	64	36	0.54
*ITGA2*	rs1062535	87	114	40	60	40	84	109	27	63	37	0.343
*PLA2G7*	rs1051931	159	75	6	82	18	150	64	6	83	17	0.795
*HMOX1*	rs2071746	74	120	47	56	44	57	115	48	52	48	0.29
*PTGS1*	rs10306114	213	28	0	94	6	195	24	1	94	6	1
*PTGS1*	rs1330344	147	85	8	79	21	119	84	17	73	27	0.044 **
*PTGS2*	rs20417	166	67	8	83	17	150	62	8	82	18	0.862
*PTGS2*	rs689466	176	58	7	85	15	163	54	3	86	14	0.638
*ADRA2A*	rs4311994	188	2	51	78	22	174	41	5	88	12	1
*9p21.3*	rs10120688	65	131	45	54	46	68	115	37	57	43	0.389
*ABCB1*	rs1045642	64	120	57	51	49	61	106	53	52	48	0.947
*PEAR1*	rs12041331	202	37	2	91	9	179	39	2	90	10	0.567

* *p*-value for comparison of alleles between the AS and AR groups; ** *p*-value < 0.05

## Data Availability

The data presented in this study are available on request from the corresponding author.

## Supplementary Materials

The following supporting information can be downloaded at: https://www.mdpi.com/article/10.3390/biomedicines10102564/s1. Figure S1: The pathway of TXA2 production and the antiplatelet effect of aspirin.; Figure S2: The biochip scheme and an example of analysis.; Table S1: Primer sequences.; Table S2: Allele-specific oligonucleotide probe sequences.; Table S3: Distribution of genetic markers in AS- and AR-groups.; Table S4: AA- and ADP-induced aggregation based on genotypes.; Model S1: ML model for aspirin resistance.

## Appendix A. Parameters of the Best-Performing Model

loss_function = ‘Logloss’
learning_rate = 0.01
Iterations = 500
depth = 10
grow_policy = ‘Lossguide’
max_leaves = 30
od_type = ‘IncToDec’
od_pval = 0.05
od_wait = 10
