# Supplementary material for: Genetic Association Study and Machine Learning to Investigate Differences in Platelet Reactivity in Patients with Acute Ischemic Stroke Treated with Aspirin
Source: Biomedicines. 2022 Oct 13;10(10):2564. doi: 10.3390/biomedicines10102564 (PMC9599820; doi:10.3390/biomedicines10102564)
Supplement: Supplementary file 1 [file biomedicines-10-02564-s001.zip › Figure S2_The biochip scheme and an example of analysis.pdf]

A

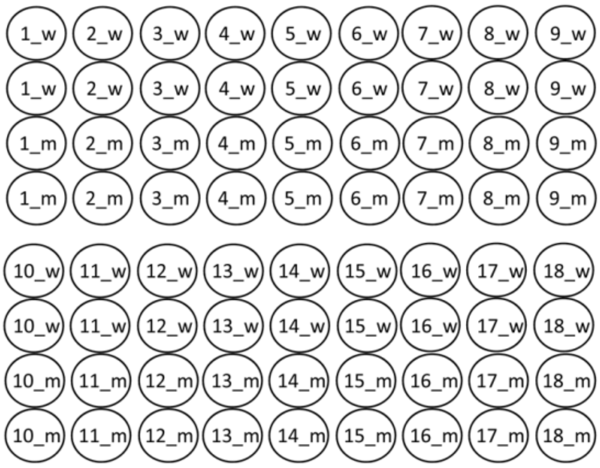

B

1_w	rs5918_T
1_m	rs5918_C
2_w	rs2243093_T
2_m	rs2243093_C
3_w	rs6065_C
3_m	rs6065_T
4_w	rs1131882_C
4_m	rs1131882_T
5_w	rs4523_C
5_m	rs4523_T
6_w	rs1126643_C
6_m	rs1126643_T
7_w	rs1062535_G
7_m	rs1062535_A
8_w	rs1051931_C
8_m	rs1051931_T
9_w	rs7756935_A
9_m	rs7756935_C

10_w	rs2071746_A
10_m	rs2071746_T
11_w	rs10306114_A
11_m	rs10306114_G
12_w	rs1330344_T
12_m	rs1330344_C
13_w	rs20417_G
13_m	rs20417_C
14_w	rs689466_T
14_m	rs689466_C
15_w	rs4311994_C
15_m	rs4311994_T
16_w	rs10120688_G
16_m	rs10120688_A
17_w	rs1045642_T
17_m	rs1045642_C
18_w	rs12041331_G
18_m	rs12041331_A

C

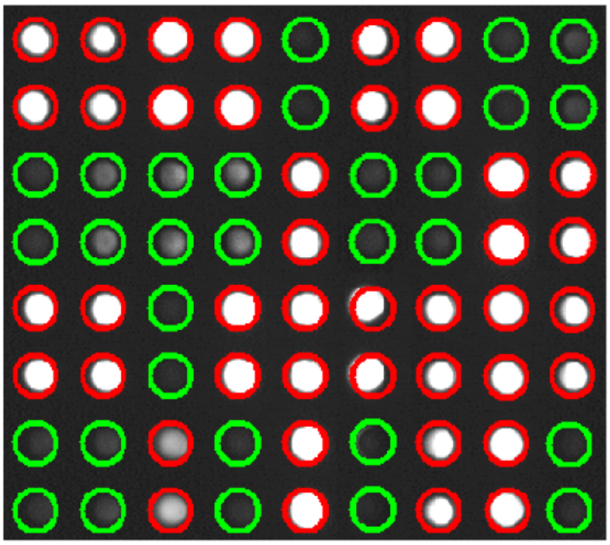

D

ITGB3 _rs5918	TT
GPIba _rs2243093	TT
GPIba _rs6065	CC
TBXA2R _rs1131882	CC
TBXA2R _rs4523	TT
ITGA2 _rs1126643	TT
ITGA2 _rs1062535	GG
PLA2G7 _rs1051931	TT
PLA2G7 _rs7756935	CC
HMOX1 _rs2071746	AA
PTGS1 _rs10306114	AA
PTGS1 _rs1330344	CC
PTGS2 _rs20417	GG
PTGS2 _rs689466	TC
ADRA2A _rs4311994	CC
9p21.3 _rs10120688	GA
MDR _rs1045642	TC
PEAR1 _rs12041331	GG

Figure S2. The biochip scheme and an example of analysis. A - the biochip scheme, B - symbols of oligonucleotide probes in biochip cells, C - an example of analysis, D – determined genotypes
