# Supplementary figures and images for: Genetic Association Study and Machine Learning to Investigate Differences in Platelet Reactivity in Patients with Acute Ischemic Stroke Treated with Aspirin
Source: Biomedicines. 2022 Oct 13;10(10):2564. doi: 10.3390/biomedicines10102564 (PMC9599820; doi:10.3390/biomedicines10102564)

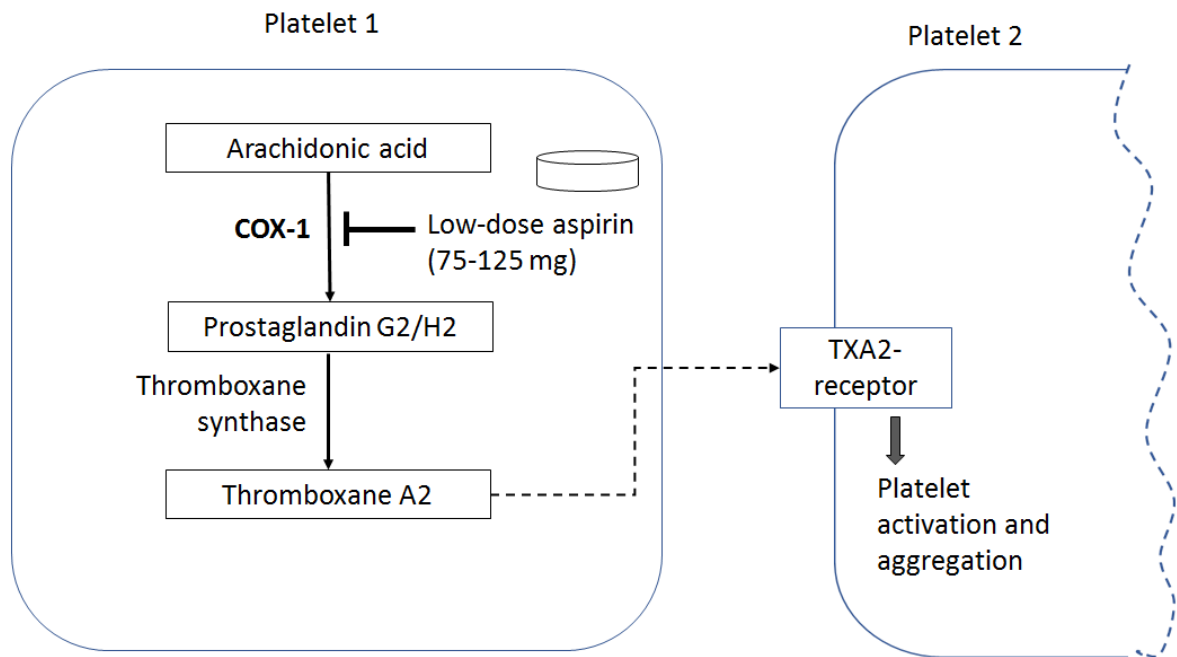

**Figure S1.** The pathway of TXA<sub>2</sub> production and the antiplatelet effect of aspirin

Supplement: Supplementary file 1 [file biomedicines-10-02564-s001.zip › Figure S1_The pathway of TXA2 production and the point of aspirin action.pdf]
